# Perspectives on Humanizing and Liberatory Qualitative Research with Racially/Ethnically Minoritized Youth

**DOI:** 10.3390/healthcare9101317

**Published:** 2021-10-01

**Authors:** Shawn S. Savage, Royel M. Johnson, Alex J. Kenney, DaVonti’ D. Haynes

**Affiliations:** 1 Department of Educational Leadership, Watson College of Education, The University of North Carolina Wilmington, Wilmington, NC 28403, USA; 2 Department of Education Policy Studies, College of Education, The Pennsylvania State University, State College, PA 16801, USA; rjohnson@psu.edu (R.M.J.); ajk5460@psu.edu (A.J.K.); 3 School of Social Work, College of Public Health, Temple University, Philadelphia, PA 19122, USA; davonti.haynes@temple.edu

**Keywords:** humanizing research, liberatory research, emotional health, minoritized youth

## Abstract

The visible impacts of COVID-19 and racial injustice have resulted in renewed funding commitments and research within minoritized communities. However, this work is too often anchored in deficit and damage-centered research approaches and practices. In this brief, we call on the qualitative research community to reframe their perspectives and terminate harmful, pain-driven research. We underscore the importance of humanizing and liberatory approaches to research with youth who are racially/ethnically minoritized. Specifically, we contend that the emotional health and overall well-being of youth are impacted by the approaches employed by researchers and the experiences racially/ethnically minoritized youth have with research. Thus, we offer specific anti-oppressive strategies and recommendations for qualitative researchers to consider in their work with racial/ethnically minoritized youth and communities.

## 1. Introduction

Qualitative research is laced with deficit and damage-centered perspectives about racially/ethnically minoritized youth (we use “minoritized” to emphasize that groups are subjected to oppression and subordination that relegate them to minority status) that prioritize documenting their pain and brokenness [1], often in exchange for material or political gain. For instance, research on Black college students too often centers solely on the racialized violence they experience at white-serving institutions (WSIs). In these studies, Black students are invited to speak, but only of their pain [2]. To be sure, shedding light on the ways in which WSIs inflict physical, emotional, and psychological harm on Black and other racially/ethnically minoritized students is important. However, it is only one aspect of their educational and lived experience. Tuck and Yang [3] remind us that “pain narratives are always incomplete” (p. 231).

Over a decade ago, Eve Tuck [1] urged scholars to place a moratorium on damage-centered research. This prescient call to action takes on heightened importance for the qualitative research community today given the current social, political, and racial climates. Data indicate that the COVID-19 pandemic has disparately impacted Black, Latinx, and Native American communities [4]. Moreover, a resurgence of White supremacy, spearheaded by former President Donald Trump, has provoked wanton racism, violence, and police brutality against racially/ethnically minoritized communities. As a result, prominent foundations and agencies have called for—and continue to solicit—research that examines how the pandemic and the onslaught of racial violence exacerbate inequities (e.g., experiences, trajectories, and outcomes) among racially/ethnically minoritized youth. While this approach—that is, documenting how oppressive structures coalesce in the dispossession of racially/ethnically minoritized youth—may seem promising, it is pathologizing in that it singularly defines a group through its oppression [1].

To challenge one-dimensional depictions of racially/ethnically minoritized youth as broken, hopeless, and inherently deficient, it is important that qualitative researchers broaden the aperture in their work to also capture the hopes, desires, agency, and resistance of racially/ethnically minoritized groups. Accounting for such complexity and contradictions is necessary, especially in this current moment, to provide fuller representations of their lived experience. Heeding Tuck’s [1] call to action from over a decade ago, we call on the research community, yet again, and specifically qualitative researchers, to permanently discontinue damage-centered research and to take action to remediate oppressive research ideologies and practices that dehumanize racially/ethnically minoritized youth and relegate them to the margins. Our work coalesces with and extends that of other scholars, e.g., [5,6,7], committed to humanizing and liberatory research [8,9].

We define humanizing research as work that is predicated on the nurturing of relationships of care and dignity among researchers and participants [10]. There is no single way to humanize research [11]; however, humanizing perspectives can frame elemental research practices, such as fieldwork, participant observation, and interviewing. By employing humanizing perspectives, researchers can work to unlearn practices of engaging participants as sites of exploitation and, instead, consider ways to give to and learn from participants. Liberatory research is freedom-focused and seeks to acknowledge the individual and the communal worth of racially/ethnically minoritized students [12]. Liberatory research can emancipate racially/ethnically minoritized populations from prescribed narratives of pain and damage in the literature and in society at large. These approaches are critically important for any research, especially with racially/ethnically minoritized youth and other systemically minoritized groups. In the following section, we identify several promising strategies for approaching and conceptualizing humanizing and liberatory educational research.

## 2. Approaching and Conceptualizing Humanizing and Liberatory Qualitative Research with Racially/Ethnically Minoritized Youth

Scholars committed to humanizing and liberatory research should work with racially/ethnically minoritized communities to frame and conceptualize their work. Indeed, the voices and experiences of youth are central to the formulation of educational issues or “problems” that are deemed worthy of scholarly attention. In this way, racially/ethnically minoritized youth must be regarded as “knowers” and thought partners in research that affects them. By consulting and partnering with participants, researchers can acknowledge, situate, and engage with participants’ humanity, rather than treating them as “subjects” upon whom research is conducted. Anti-oppressive qualitative scholars can solicit feedback from these research partners about the kinds of questions they might have, the kinds of answers they think institutional researchers should seek, and the kinds of data they wish to keep within the confines of the research process and not have reported out or published. Such an approach can help to mitigate extractive unidirectional deficit-oriented questions and damage-centered research that are devoid of participants’ human complexity. As Edwards and colleagues note, researchers must make more concerted efforts to regard their participants “as human beings with whom we might develop strong and lasting bonds” ([13] p. 433).

It is also important that the research questions that animate one’s work are strengths-based. For instance, as Harper [14] illuminates, a deficit-based approach to exploring the experiences of college students designated as low-income, for instance, would ask questions such as, “Why are low-income students retained at lower rates than their counterparts?” or, “Why are low-income students less engaged in the campus community?” In contrast, a strengths-based approach may reframe those questions to obtain new information about how students designated as low-income defy those claims and explore how they find success within their campus community. Using strengths-based approaches, the research may ask, “What institutional services did low-income students identify as being most effective in supporting them during their collegiate experience?” or, “What types of activities are low-income students most engaged in on campus?” Such an approach should not be perceived as ignoring the problems and struggles of the community, however. Instead, it is making a conscious effort to regulate the extent of attention being placed on the perceived deficits of the community and focus more on a strengths-based and desire-centered approach [15].

Engaging in liberatory and humanizing research also means interrogating and dismantling the dominant logics and paradigms that govern and shape how we engage research with racially/ethnically minoritized communities. One such institutional structure we should problematize is institutional review boards (IRBs) for research with human “subjects.” The 1979 Belmont report established cognizance of vulnerable populations as a cornerstone of ethical research [16]. To this end, certain populations, such as those in the criminal punishment system or those in foster care, have been deemed “vulnerable” and thus require additional protections during research. Stewart [17] problematizes the notion of vulnerability as it presumes the inherent incapacity to give or withhold informed consent to participate in a study. Instead, populations are “vulnerablized” in research, referring to the process by which racially/ethnically minoritized and other minoritized groups are made vulnerable because of oppressive, exploitive, and deceitful research practices and policies. While touted as an “apparent good, resulting from gross historical abuses of participants’ rights of research” [17] (p. 5), IRBs are not the arbiters for humanizing and liberatory research. Nor are they off the hook for their complicity in perpetuating oppressive and damage-centered research on racially/ethnically minoritized youth and others. For instance, Stewart [17] notes that populations deemed vulnerable “receive no protection from the mindset of researchers whose studies do not disrupt oppressive constructs and norms” (p. 6).

Qualitative researchers must adopt their own internal protocols for assessing and addressing the various forms of harm that one might inflict on participants during the research process. Important questions for anti-oppressive researchers to consider include:

1. What deficit- and damage-centered assumptions do I hold about my participants?
2. What are the sources of these assumptions?
3. In what ways have these assumptions informed my approach (e.g., problem formulation, research design, theoretical orientation) to the study?
4. What steps can I take to remediate such assumptions and reduce harm in the research process?
5. What is my personal and professional responsibility to the populations and communities I aim to study?
6. In what ways have I centered and engaged racially/ethnically minoritized youth in the research process beyond data collection?
7. How will I honor and respect the stories and other forms of data that are shared with me through the research process?
8. How will I use what is generated from this research to improve the material conditions of the population I am studying?

It is important that qualitative researchers develop a critical consciousness and begin to interrogate the dominant logics that undergird canonical approaches to their work. Conducting humanizing and liberatory educational research requires a commitment to learning, unlearning, and cultural humility. It means framing and conceptualizing research with the communities one studies; centering their experiential knowledge; resisting archaic theories of change that are predicated on illustrating one’s brokenness and pain; practicing critical reflexivity; prioritizing the needs and well-being of participants over those of the researcher and the academy; and anchoring one’s work in critical and transformative epistemologies that focus on changing and dismantling oppressive structures and systems.

## 3. Humanizing and Liberatory Research Designs

In addition to framing and conceptualizing qualitative research in anti-oppressive ways, one’s research design and methods must also reflect humanizing and liberatory commitments. To be sure, the recommendations presented in this section are not meant to be exhaustive. Instead, we hope that the few examples presented here serve as a useful starting point for qualitative researchers committed to honoring and respecting the humanity, agency, and desires of racially/ethnically minoritized youth and their communities.

Youth participatory action research, or YPAR [18,19], is one common methodological approach, especially when anchored in critical or transformative epistemological traditions, that has enormous potential for embodying humanizing and liberatory research. So too does community-based participatory research (CBPR), which is especially common in health fields [20,21]. The amenability of these research methodologies and traditions to humanizing and liberatory perspectives is particularly evident in the participatory action research with people who are minoritized because of (dis)ability (see [6,7]). And there is a broader body of work that examines the positive impacts of liberatory approaches to issues related to health in the literature on public and community engagement in research (see for example, [8,22,23]). Whether immuno-compromised, economically disenfranchised, impacted by foster care, LGBTQIA+, immigrant, DACAmented, or justice-involved, minoritized youth must be given space to be actively engaged in the research process; the centering of their voices and desires is “ethically necessary” ([10] p. 1). To be sure, it also enhances the validity of the research. In these ways, institutional researchers are partners in bringing visibility, advancing material systemic changes, and celebrating the agency, competence, well-being, beauty, and brilliance of youth. This interrupts the pimp-to-publish pipeline culture that often characterizes research with communities that are systemically harmed. Like a pimp, researchers often use participants to advance their publication and career goals without also being attentive to the broader well-being of participants.

We also propose the use of what we will here coin as “youth practitioner research” (YPR); that is, research with and by youth who are themselves not merely members of specific groups that are minoritized, but who are actively engaged in liberatory praxis for their own and their fellow group members’ benefit. Practically, we can consider various advocacy groups where youth are deeply involved in liberatory work on the ground and online, including organizing, strategizing, educating, and advocating for their various communities and others who are systemically harmed. These groups include immigrant rights groups, community organizations, health advocacy groups, LGBTQIA+ advocates, and those who are working for the transformation of the criminal punishment system on behalf of justice-involved youth. These “practitioners”—in their own rights—could prove immeasurably critical to humanizing research. By engaging in these kinds of research with (rather than on or about) racially/ethnically minoritized youth, traditional (i.e., institutional) researchers would contribute to shifting from deficit-oriented questions [24], and damage-centered research [1] towards more humanizing and liberatory research designs.

We acknowledge that the methodologies we have highlighted here are most frequently used within a qualitative research paradigm. However, we recognize that researchers committed to disrupting and reframing their research praxis for humanizing and liberatory ends will see our perspectives as suggestions that could inform the design of quantitative and mixed methods approaches as well. Recent developments, and the increasing proliferation of QuantCrit, for example, illustrate that critical approaches to research do not preclude quantitative inquiry, just as DisCrit has enabled a more focused attention to disability research through critical lenses. Therefore, as we previously underscored, we offer these perspectives for humanizing and liberatory research as suggestive rather than prescriptive. We argue that even within a quantitative paradigm, researchers who utilize humanizing and liberatory perspectives would study the experiences of Black and Brown communities without using White participants as the norm, or necessarily including them in the analyses at all. Those research design decisions can be easily made because a strengths-based approach—informed by the perspectives we advance in this article—underscores that the communities being studied are sufficient on their own. We therefore encourage quantitative researchers to explore the ways in which our perspectives here may be infused into their research as well.

## 4. A Multi-Tiered Imperative

This brief report shared perspectives, approaches, and research designs for humanizing and liberatory qualitative research, particularly with racially/ethnically minoritized youth. In so doing, we have emphasized the critical importance of terminating the use of damage-centered research approaches, and reframing research with racially/ethnically minoritized youth. In this section, we offer two tables to highlight the nature and impact of damage-oriented research versus humanizing and liberatory research, and then discuss further issues for consideration. Specifically, Table 1 and Table 2 underscore some of the ways harm and health are enacted through research.

Against the background of the contrasting impacts captured in the tables, we close this brief with some additional considerations for how qualitative researchers might further center humanizing and liberatory perspectives by activating specific moves in their work. But we do acknowledge that although individual researchers’ roles are important, the research infrastructures of institutions and their relationships with communities are also very significant. Consequently, the recommendations with which we close, are multi-tiered.

First, we recommend that individual qualitative researchers examine and expand the roles of racially/ethnically minoritized youth in qualitative research projects. For example, one recommendation is the adoption of youth or community advisory boards (see [25]), which is a common practice in public health research, although few education scholars have adopted this practice as well (see [26,27]). This approach is a promising strategy for developing trust, fostering understanding, and promoting meaningful engagement with youth and community stakeholders. It can also help ensure that the research one is proposing is relevant and important to the communities one is seeking access to for study. For instance, advisory boards may formulate (or give feedback on) research questions and interview protocols, collaborate on data analysis, offer insights into endogenous social and cultural contexts and meanings, coauthor manuscripts from the research, and disseminate study findings.

Second, we also recommend that qualitative researchers work within and across institutions to push for systemic changes in the research processes of higher education institutions and other research entities. Importantly, researchers should advocate for the transformation of institutional policies and practices that shape how we conduct and approach research with (racially/ethnically) minoritized communities. As mentioned in an earlier section, IRBs should not be absolved from their complicity in fostering oppressive research. Scholars ought to advocate for new screening criteria and metrics that are anchored in humanizing and liberatory perspectives. That community members and stakeholders are not involved in making determinations about the level of risks associated with proposed research is problematic. Might we imagine IRBs that include compensated board roles for community members and youth who might work with institutions to imagine new review criteria? Similarly, might we revise our IRB processes to require that research protocols meet specific anti-oppressive criteria? Actions at this tier would further communicate and demonstrate a commitment to anti-oppression as well as to humanizing and liberatory research.

Third, both the work of individual qualitative researchers and institutional changes with IRB, for instance, must also be complemented with broader efforts to change the research relationships that researchers, and institutions have with communities. Researchers and their institutions might better serve racially/ethnically minoritized youth through more concrete relational commitments. For example, what grants and university funds might be channeled to sustain relationships with communities beyond when participants are needed for studies? How can sustained relationships with minoritized youth and communities be institutionalized? Furthermore, the research ideation phase does not merely have to be the purview of the university-based researcher. So, how might universities create spaces in which youth within a given community might be able to initiate and propose research ideas that later get supported to completion by the research expertise and financial leverage of universities and other research entities?

We contend that these recommendations and considerations underscore the multi-tiered imperative of ensuring the termination of research vested in damage, and a reconstitution of robust qualitative research that ensures it is sustainably humanizing and liberatory. The conceptualizations, approaches, research designs, and considerations we have discussed are rife with material promise. Their generativity bodes well for qualitative research. Given recent calls and funding for research with communities and populations severely impacted by COVID-19 and racial injustice, the perspectives we offer become even more necessary—particularly in research involving racially/ethnically (and otherwise) minoritized youth. Their health and well-being—emotional and physical—are, in many ways, impacted by those of us engaging in research. And their health and well-being demand more humanizing and liberatory approaches from all of us. Therefore, let us end research that engages in and perpetuates damage, so that we can sustain humanizing and liberatory possibilities with racially/ethnically minoritized youth.

## Figures and Tables

**Table 1:** Damage and Deficit- Centered Qualitative Research.

Pain-driven Research	Stigmatizes and deficitizes participants and communities and positions them as problems Propels myths about minoritized communities and populations Do not recognize participants’ human agency and resiliency Lacks involvement of participants in research design, execution, and analyses Engenders feelings of embarrassment, betrayal, disappointment, and inadequacy that may in turn lead to mental, emotional, and physical ill-health and negatively impact well-being

**Table 2:** Humanizing and Liberatory Qualitative Research.

Humanizing and Liberatory Research	Centralizes and celebrates participants as complex and as sources of knowledge and pride Debunks negative perceptions about racially/ethnically minoritized communities and populations Recognizes human agency and reveals the strengths of communities and populations Engage participants in its research design, implementation, and analyses Engenders feelings of celebration, affirmation, beingness and belongingness, that can lead to positive mental, emotional, and physical well-being

## Data Availability

Not applicable.
